# Supplementary figures and images for: Transcriptome analysis of the differences between two kinds of Cassia nomame germplasm resources
Source: PeerJ. 2025 Oct 30;13:e20261. doi: 10.7717/peerj.20261 (PMC12579854; doi:10.7717/peerj.20261)

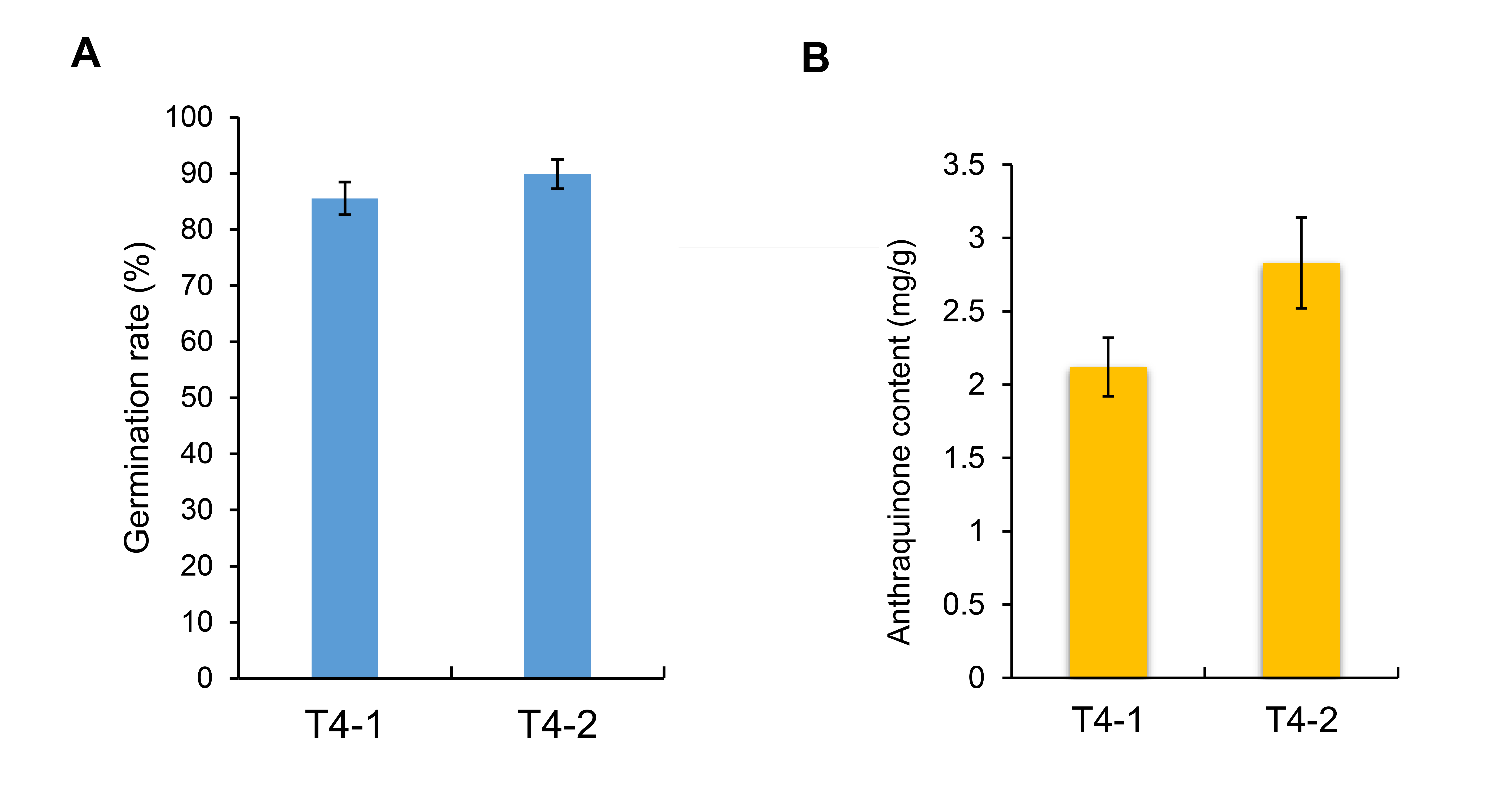

Supplement: Supplemental Information 1 [file peerj-13-20261-s001.jpg]

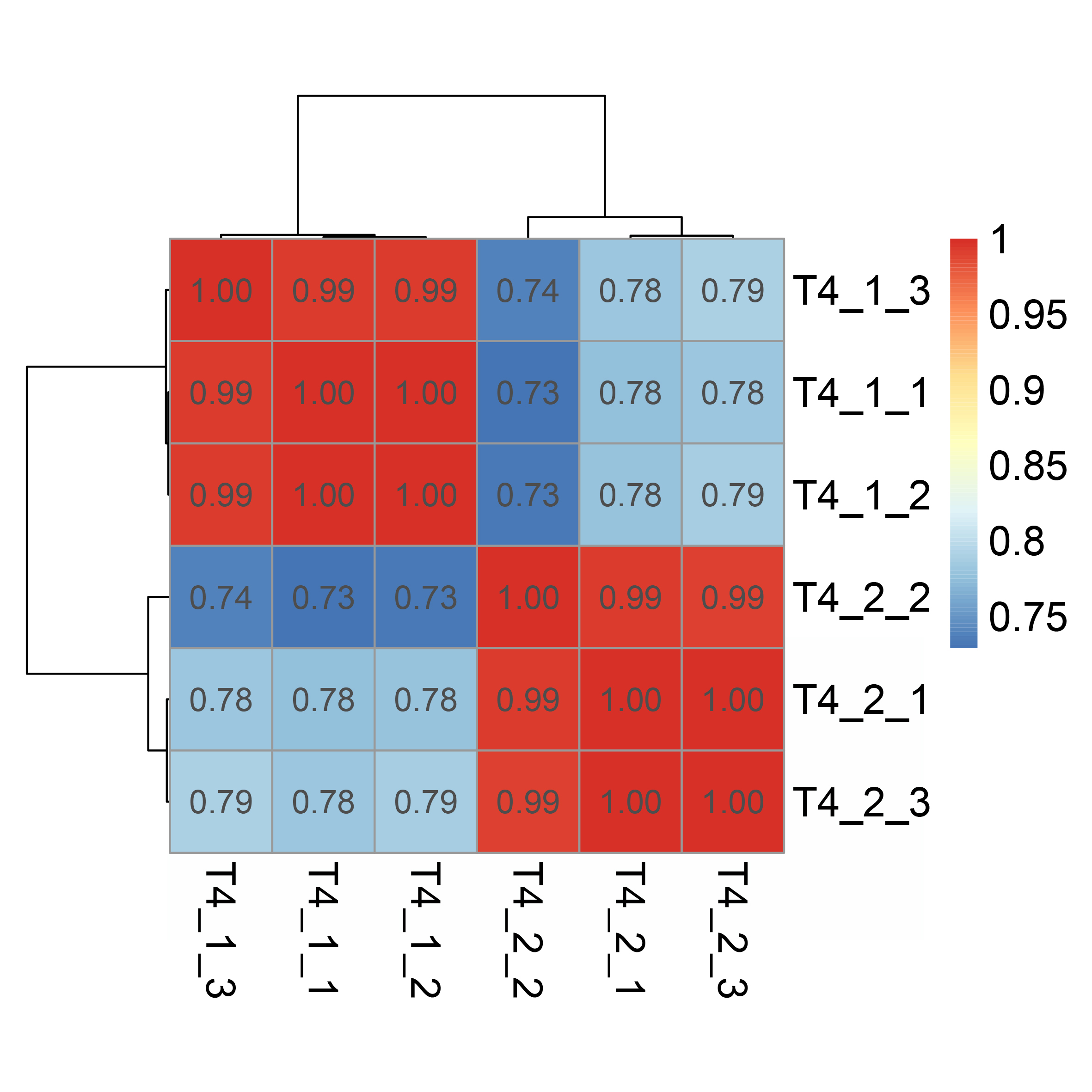

Supplement: Supplemental Information 2 [file peerj-13-20261-s002.jpg]

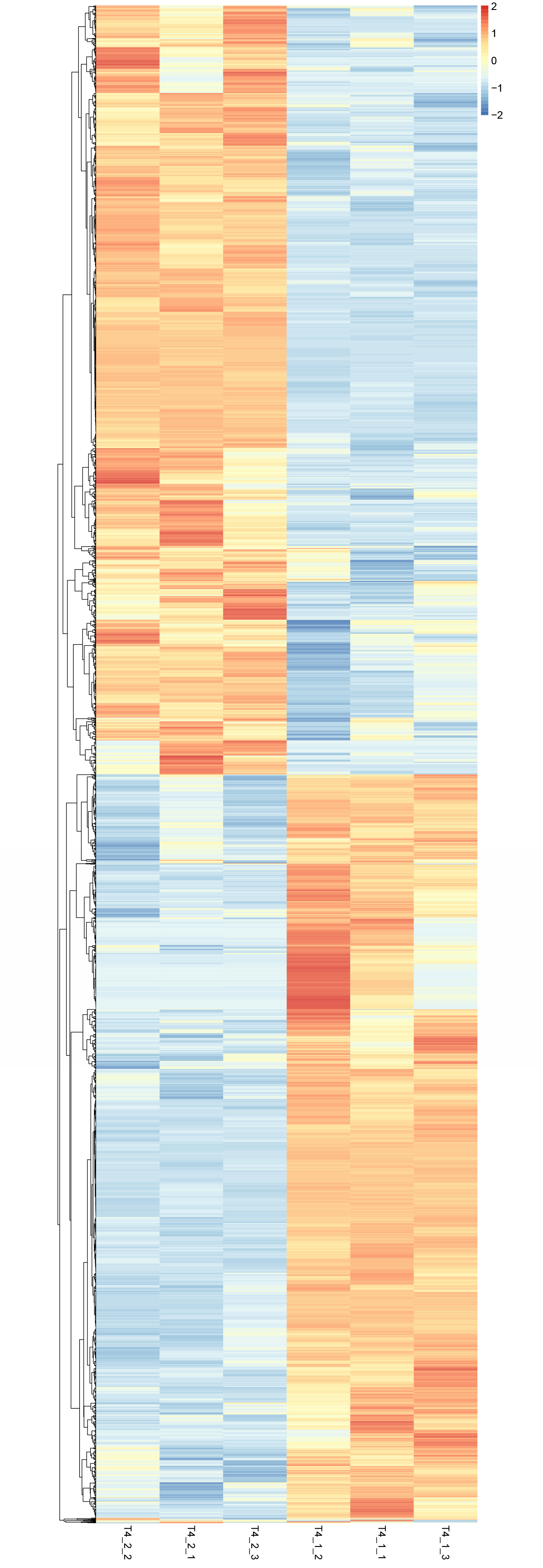

Supplement: Supplemental Information 3 — Based on the Z-score strategy, the expression level is represented by values ranging from −2 to 2, which indicates low to high. [file peerj-13-20261-s003.jpg]

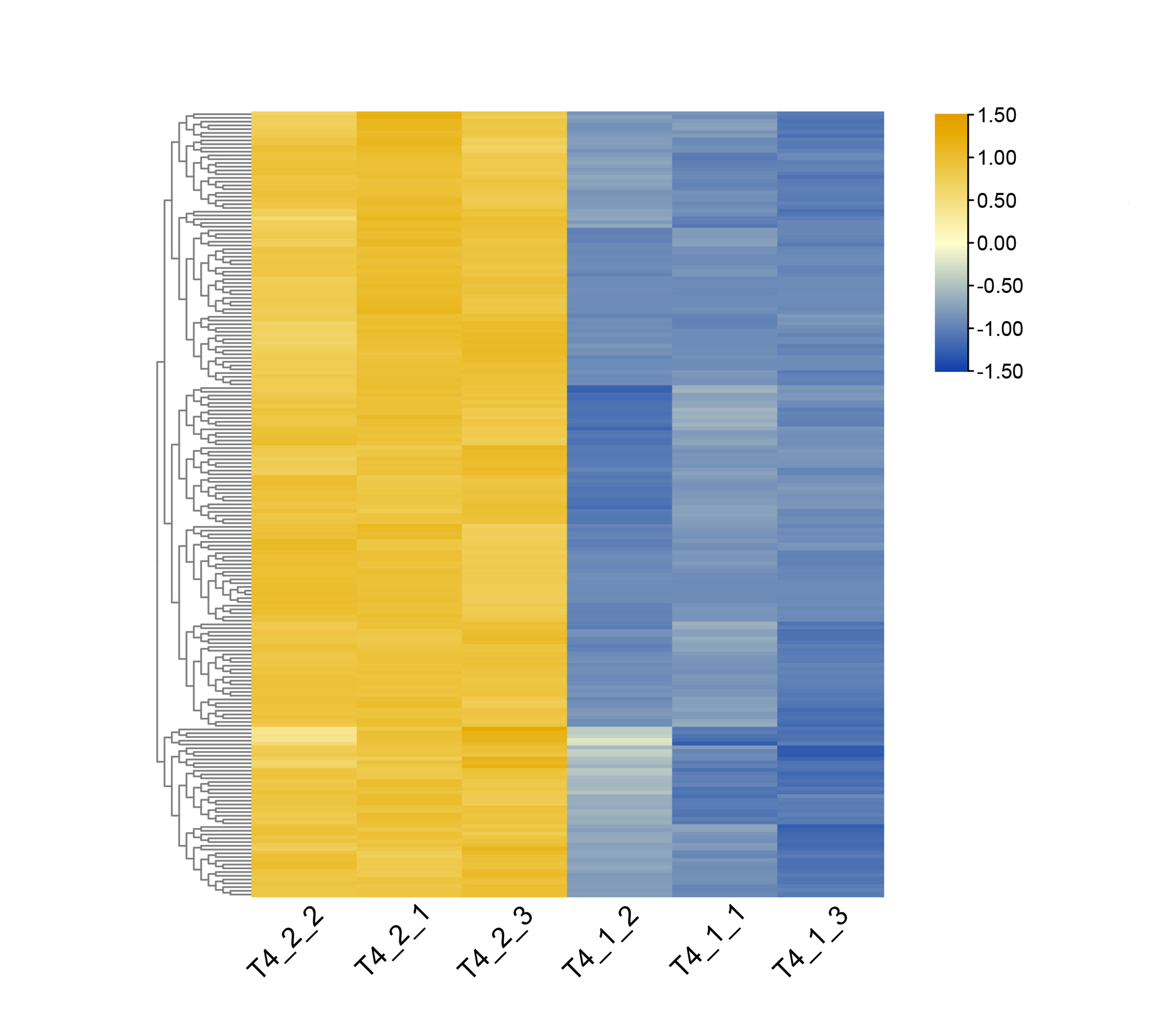

Supplement: Supplemental Information 4 — Based on the Z-score strategy, the expression level is represented by values ranging from −2 to 2, which indicates low to high [file peerj-13-20261-s004.jpg]
